# Commentary: Mechanisms of kwashiorkor-associated immune suppression: Insights from human, mouse, and pig studies

**DOI:** 10.3389/fimmu.2022.959465

**Published:** 2022-07-25

**Authors:** Gerard Bryan Gonzales, Claire D. Bourke, Jonathan P. Sturgeon, James M. Njunge, Ruairi C. Robertson, Paul M. Kelly, James A. Berkley

**Affiliations:** ^1^ Nutrition, Metabolism and Genomics Group, Division of Human Nutrition and Health, Wageningen University and Research, Wageningen, Netherlands; ^2^ Zvitambo Institute for Maternal and Child Health Research, Harare, Zimbabwe; ^3^ Blizard Institute, Queen Mary University of London, London, United Kingdom; ^4^ The Childhood Acute Illness & Nutrition (CHAIN) Network, Nairobi, Kenya; ^5^ Kenya Medical Research Institute (KEMRI) - Wellcome Trust Research Programme, Nairobi, Kenya; ^6^ Tropical Gastroenterology and Nutrition Group, University of Zambia, Lusaka, Zambia; ^7^ Nuffield Department of Medicine, Centre for Tropical Medicine & Global Health, University of Oxford, Oxford, United Kingdom

**Keywords:** kwashiorkor, immune, severe malnutrition, marasmus, edematous malnutrition

## Introduction

Derangement in functional immunity is a characteristic of children with severe malnutrition (SM). Deaths among children with SM are principally from infections, and understanding their pathophysiology to develop effective treatment strategies is essential to improving outcomes. Several reviews have discussed the state of knowledge of SM immunology (1–3). However, differences in immune function between kwashiorkor and wasting have not been adequately documented.

We were delighted to see a review of kwashiorkor-associated immune suppression, as there are very few reports that have studied kwashiorkor-specific immunological changes, especially in clinical settings. However, we believe that the review of Michael et al. (4) on kwashiorkor-associated immune function contains several oversimplifications and extrapolations of results given the recognized heterogeneity and power limitations of existing immunological assessments in SM (5). We believe that clarity in reporting results from clinical studies (also highlighted by literature reviews cited by Michael et al.) and in how they compare to experimental models is essential to add value to the wider field of malnutrition-related immunology. Therefore, while we applaud the authors for their work, we are concerned about several aspects of their recent review for reasons we discussed in this commentary.

## Discussion

Firstly, the idea that reduced protein intake leads to hypoalbuminemia, which decreases oncotic pressur leading to edema in kwashiorkor, is widely believed, but this is an oversimplification which is not supported by strong evidence. In a recent study, we found that hypoalbuminemia is associated but alone was insufficient to explain edema in kwashiorkor (6). Other factors apart from hypoalbuminemia and low protein intake must develop kwashiorkor (7), such as extracellular matrix (ECM) degradation and lymphatic damage (6). Moreover, it was previously shown that edema in children with kwashiorkor resolved even when they were treated with a protein-deficient diet (8), and despite a small increase in serum albumin concentration among children whose oedema resolved or improved, serum albumin concentrations remained far below clinically recognized norms in children (6, 9). It is important to emphasize that oversimplification of kwashiorkor etiology (presented in the review text and Figure 2 but unsupported by empirical evidence) can result in ineffective treatment strategies.

Secondly, some of the immune function effects stated in the review are not borne out by the cited references. It is tempting to suggest that kwashiorkor is characterized by a “profound impaired immune function,” as the authors claim. For instance, diseases with heightened inflammation are associated with ECM degradation, and the degradation of the ECM is linked to immune function (10). However, the papers cited did not study kwashiorkor specifically. For instance, the paper by Hughes et al. (11) involved both wasting and kwashiorkor. Half of these children also had HIV, and most likely had other infections, which resulted in their hospital admission. The analysis by Hughes et al. was controlled for edema; thus, no kwashiorkor-specific estimates were presented in the paper. The table given below lists statements about kwashiorkor-associated immunological characteristics in the review by Michael et al. that are not supported by the cited references.

Evaluation of claims regarding kwashiorkor-associated immunological changes.

**Table:** 

**Claim**	**Reference** **cited**	**What the referenced study** **did or observed**
“*Zambian children with kwashiorkor had normal numbers of white blood cells (WBCs), however, although the numbers of monocyte-derived DCs were reduced in their peripheral blood. The kwashiorkor-induced impairments were rescued following intervention using a protein-sufficient diet*”	(11)	Study involved children with severe malnutrition, but no kwashiorkor-specific estimates were presented in the paper. N = 57 kwashiorkor; 24 marasmus; and 39 had HIV
“*Fas (CD95/apoptosis antigen 1), a gene that signals to initiate apoptosis, is highly expressed in neutrophils, monocytes, and lymphocytes in kwashiorkor children indicative of impaired regulation of immunity and lymphoid homeostasis”* “*this study showed that the expression of this marker was reduced following feeding a protein-sufficient diet, suggesting that the life cycle of WBCs is limited in kwashiorkor conditions*”	(12)	CD95 expression of neutrophil and lymphocyte was found higher in kwashiorkor than healthy controls without differences in monocyte CD95 gene expression. Neither apoptosis nor life-span of the cell types were assessed to support claims for differential ‘life cycle of WBC’ by SM nor kwashiorkor specifically. There were no differences in CD95 expression in neutrophils, lymphocytes and monocytes between kwashiorkor and marasmus. This indicates that CD95 gene expression is generally affected by malnutrition, not specifically to kwashiorkor.
“*Children with kwashiorkor and/or respiratory/gastrointestinal infections had increased apoptotic T cells, increased Fas (CD95) expression, and reduced levels of IL-7/IL-7 Rα and expressed inhibitory receptor-programmed death (PD-1) expression on T cells*”	(13)	The study included children with severe malnutrition, but no kwashiorkor-specific estimates were presented in the paper. N = 10 kwashiorkor; 19 marasmus
“*Decreased numbers of B lymphocytes in kwashiorkor children with gastrointestinal or respiratory infections compared with well-nourished children having similar infections”*	(14)	The study involved children with severe malnutrition, but no kwashiorkor-specific estimates were presented in the paper. N = 3 kwashiorkor; 7 marasmus
“*increased risk of Gram-negative bacteriemia in hospitalized kwashiorkor children*”	(15)	Our study involved all children under the age of 13 years who were admitted to a hospital. No kwashiorkor-specific estimates were presented in the paper.
“*Studies from Bangladeshi children revealed that the kwashiorkor-associated faecal microbiota was significantly less diverse (immature) compared with that of age-matched healthy children. However, this condition was reversible, and the microbiome composition has been restored to the diverse (mature) phenotype when these kwashiorkor children were given RUTF and treated with antibiotics”*	(16)	Study involved children with severe malnutrition, but no kwashiorkor-specific estimates were presented in the paper.
“*Kwashiorkor children (6 to 59 months old) were treated for 7 days with cefdinir, amoxicillin, or placebo in combination with RUTF, showed that children that received RUTF and antibiotics had accelerated weight gain, decreased mortality rates, and increased recovery rates than those who received placebo*”	(17)	Study involved children with severe malnutrition, but no kwashiorkor-specific estimates were presented in the paper.
*“Clinical studies in children have revealed a relationship between lower seroconversion rates associated with oral vaccines and kwashiorkor”*	(18, 19)	The cited studies involved patients with inflammatory bowel disease. No kwashiorkor patients were involved in the cited studies.

The review also contains a section about micronutrient deficiencies specific to kwashiorkor. However, none of the micronutrient literature presented is specific to kwashiorkor, much less to wasting. Furthermore, the review highlighted the impact of kwashiorkor on infection and vaccination in the gnotobiotic piglet model, which may not be translational to human kwashiorkor, as discussed below. More clinical evidence in humans is needed.

The authors use the evidence they gathered in this review to justify gnotobiotic pigs as better models of kwashiorkor than mouse models because the pig models replicate the immune function found in human kwashiorkor better than mice. However, as above, there is no strong evidence for kwashiorkor-specific immune changes. Unlike in animal models, the clinical heterogeneity inherent to SM renders many existing studies underpowered or unable to distinguish from acute infection to draw conclusions on immune function in human SM per se, let alone in kwashiorkor specifically. We agree that pig models may offer a valuable opportunity for immunological studies (2), but disagree that there is sufficient evidence to use immunological effects to validate these models.

Finally, we agree with the authors that the mouse models of kwashiorkor, which have attracted the most citations, lack face validity (2). Most importantly, the mouse model proposed by Smith et al. (20) did not develop edema, which is pathognomonic of kwashiorkor. In contrast, the pig model of the authors developed generalized edema, which is more indicative of kwashiorkor. Upon reviewing the pig model development, we noticed that the phenotype appeared when nursing piglets were fed with Parmalat (bovine whole milk containing 3.3% protein, 3.3% fat, and 5% carbohydrates) mixed with sterile water (50:50 v/v), resulting in 50% less protein (21). Control pigs were fed 100% Parmalat. This model raises two questions. First, the malnourished pigs received 50% less of all constituent macronutrients rather than reduced protein alone, making it difficult to conclude that the phenotype observed is solely due to protein deficiency. Second, kwashiorkor in children peaks at around 2 years of age, often after weaning. The neonatal gnotobiotic pigs were fed a diet deficient in macronutrients before 4 days of age. Thus, these porcine models may have different intestinal development than children with kwashiorkor, and the cause of the edema may relate to immature intestinal development or other causes. Other non-kwashiorkor pathologies in children with edema have been described by Golden (7).

In summary, the review highlighted interesting immune features in children with severe malnutrition but did not specifically describe the immune function in kwashiorkor. Hence, the reported similarities between the immune function of children with kwashiorkor and gnotobiotic pig models cannot be used to establish the face validity of the porcine model. Kwashiorkor-specific studies on immune function are still lacking, providing an opportunity for further translational research. Rather than viewing experimental models as the “only alternative to clinical studies,” we regard insights from clinical and translational immunology studies as essential to achieve the goal of the authors of carefully selecting appropriate, evidence-based mechanistic and pre-clinical models that can support therapeutic interventions for SM.

## Author Contributions

GBG wrote the initial draft. All authors listed have made a substantial, direct, and intellectual contribution to the work and approved it for publication.

## Funding

CDB is supported by the joint Wellcome Trust and Royal Society Grant (206225/Z/17/Z). JPS (220566/Z/20/Z), JMN (222967/Z/21/Z), and RCR (206455/Z/17/Z) are supported by the Wellcome Trust. JAB is supported by MRC/DFID/Wellcome Trust Joint Global Health Trials scheme (MR/M007367/1) and the Bill and Melinda Gates Foundation (OPP1131320).

## Conflict of interest

The authors declare that the research was conducted in the absence of any commercial or financial relationships that could be construed as a potential conflict of interest.

## Publisher’s note

All claims expressed in this article are solely those of the authors and do not necessarily represent those of their affiliated organizations, or those of the publisher, the editors and the reviewers. Any product that may be evaluated in this article, or claim that may be made by its manufacturer, is not guaranteed or endorsed by the publisher.
